# Anti-Tumor Effects of Astaxanthin by Inhibition of the Expression of STAT3 in Prostate Cancer

**DOI:** 10.3390/md18080415

**Published:** 2020-08-07

**Authors:** Shao-Qian Sun, You-Xi Zhao, Si-Yu Li, Jing-Wen Qiang, Yi-Zhi Ji

**Affiliations:** Biochemical Engineering College, Beijing Union University, Beijing 100023, China; shaoqian168@sina.com (S.-Q.S.); zhaoyouxi@buu.edu.cn (Y.-X.Z.); emmalee0316@163.com (S.-Y.L.); JKstrong99@163.com (J.-W.Q.)

**Keywords:** prostate cancer, astaxanthin, STAT3, proliferation, colony formation, apoptosis, migration, invasion

## Abstract

Astaxanthin is a natural product gaining increasing attention due to its safety and anti-cancer properties. In this study, we investigated the mechanisms of the anti-cancer effects of astaxanthin on prostate cancer (PCa) cell lines using aggressive PCa DU145 cells. Also an instantaneous silenced cell line (si-STAT3) derived from DU145 and a control cell line (si-NK) were used for the MTT and colony formation assays to determine the role of astaxanthin in proliferation and colony formation abilities. Flow cytometry assays were used to detect the apoptosis of tumor cells. Migration and invasion assays detected the weakening of the respective abilities. Western blot and RT-PCR tests detected the levels of STAT3 protein and mRNA. Astaxanthin resulted in suppression of the proliferation of DU145 cells and the level of STAT3. The treatment of DU145 cells with astaxanthin decreased the cloning ability, increased the apoptosis percentage and weakened the abilities of migration and invasion of the cells. Furthermore, astaxanthin reduced the expression of STAT3 at protein and mRNA levels. The effects were enhanced when astaxanthin and si-STAT3 were combined. The results of animal experiments were consistent with the results in cells. Thus, astaxanthin inhibits the proliferation of DU145 cells by reducing the expression of STAT3.

## 1. Introduction

Prostate cancer is one of the most frequently diagnosed malignancies in men, with increasing incidence in China [1,2]. Surgery, radiotherapy, chemotherapy and endocrine therapy are curative treatment options for prostate cancer [3]. However, after receiving endocrine therapy or surgery, 80–90% of patients develop hormone resistance within 3–4 years. In patients tolerant to endocrine therapy, increasing the doses of radiotherapy and chemotherapy can, rarely, improve the treatment effect, and severe toxicity and side effects cannot be avoided [4]. In addition, whether radiotherapy or chemotherapy resistance appeared after a period of treatment was elucidated using aggressive prostate cancer cells [5]. Thus, finding a drug that effectively inhibits prostate cancer is clinically significant.

Signal transducer and activator of transcription 3 (STAT3) is one of the critical members of the signal transducer and activator of transcription family and is composed of about 770 amino acids [6,7]. STAT3 activation plays a major role in mediating cell proliferation, differentiation, invasion, metastasis, inflammation, apoptosis and other biological cell processes [8,9,10,11,12]. Some studies have shown that compared to normal cells, the 705 site of the C-amino terminal tyrosine in STAT3 is continuously and abnormally activated in about 70% of tumor cells [8,12,13]. This protein is aberrantly activated in prostate cancer and contributes to the promotion of metastatic progression [14,15]. Therefore, STAT3 plays a major role in the occurrence and development of prostate cancer by effectively blocking the activation of related pathways, which is a novel method of cancer treatment.

About 67% of anti-cancer drugs are derived from natural products or natural product derivatives [16]. Astaxanthin is a natural product that is safe and possesses anti-cancer properties. It is primarily found in marine organisms such as algae, phytoplankton and shrimp. It is the final form of carotenoid synthesis with a strong ability to quench singlet oxygen and clear free radicals [17,18,19]. Recently, many in vivo and in vitro studies have confirmed that astaxanthin inhibits the growth of various tumor cells, such as neuroblastoma, lung cancer, gastric cancer, oral cancer, colon cancer, breast cancer, bladder cancer, liver cancer and leukemia [20,21,22,23,24,25,26]. Anti-cancer effects include anti-proliferation [27], enhancing apoptosis [27], anti-oxidation [28,29], anti-inflammation [28,30], preventing migration and invasion, and so on [31]. Although the mechanisms of astaxanthin mediating anti-cancer action have not yet been fully clarified, a number of molecular targets of astaxanthin have been proposed, which may explain the anti-tumor effects of this drug, such as NF-κB, STAT3, PI3K/AKT, MAPKs, PPARγ, and so on [31,32,33,34]. However, whether astaxanthin suppresses prostate cancer through STAT3 is yet to be elucidated.

Herein, whether astaxanthin can effectively inhibit the proliferation, cloning ability, invasion and migration ability and increase the apoptosis of DU145 cells by inhibiting the expression of STAT3 and its related proteins at protein and mRNA levels is evaluated.

## 2. Results

### 2.1. Astaxanthin Inhibits the Proliferation of DU145 Cells and Reduces the Expression of STAT3

**Figure 1:**
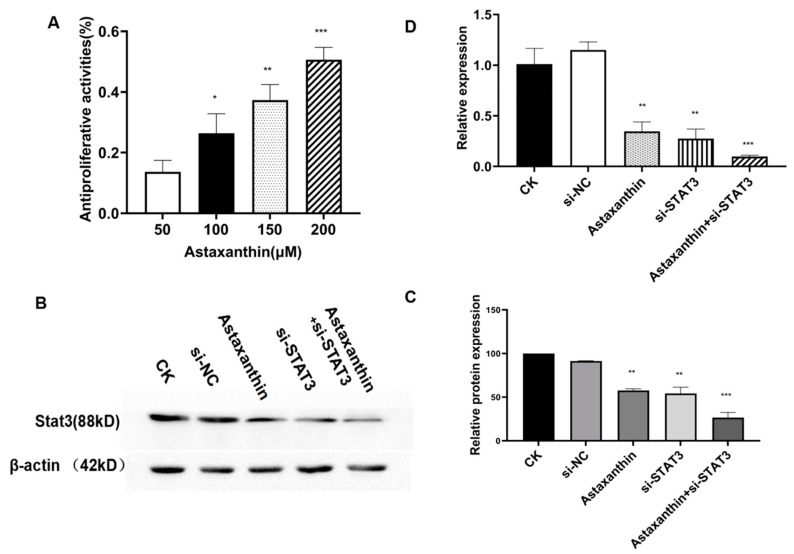
Cell proliferation assay and Western blotting using prostate cancer DU145 cells. (**A**) Cells were pretreated with 0, 50, 100 and 200 μM astaxanthin for 48 h. Proliferation was measured by MTT assay. Data are presented as the average of three experiments (±SD), * *p* < 0.05, ** *p* < 0.01, and *** p < 0.001. (**B**) DU145 cells were pretreated with 200 μM astaxanthin for 24 h. Western blotting results show that 200 μM astaxanthin reduces the level of STAT3. (**C**) Relative protein expression analysis. * p < 0.05, ** p < 0.01, and *** p < 0.001. (**D**) Relative mRNA expression analysis. ** p < 0.01, and *** p < 0.001.

To explore the effects of astaxanthin on the proliferation of DU145, we conducted the MTT assay. DU145 cells were cultured in the presence of different concentrations of astaxanthin. The results showed that astaxanthin significantly suppressed the proliferation of DU145 cells, and the inhibition of proliferation was dose-dependent. Astaxanthin suppressed cell proliferation at 50, 100 and 200% as compared to the control at the rates of 27, 38 and 50%, respectively (Figure 1A). These results indicate that astaxanthin inhibits DU145 cells, and the IC50 (half-inhibitory concentration) was <200 μM.

To explore whether astaxanthin was a potential inhibitor, we examined the expression of STAT3 in protein (Figure 1B,C) and mRNA levels (Figure 1D). The results indicated that astaxanthin is a potential inhibitor of STAT3 and downregulates its expression at both protein and mRNA levels.

### 2.2. Astaxanthin Reduces the Colony Formation Ability of DU145 Cells

CK was the blank control. The expression of STAT3 in DU145 was lowered by siRNA-STAT3, and siRNA-NC was the blank control to si-STAT3. After 48 h post-transfection, 200 μM was administered for 24 h.

**Figure 2:**
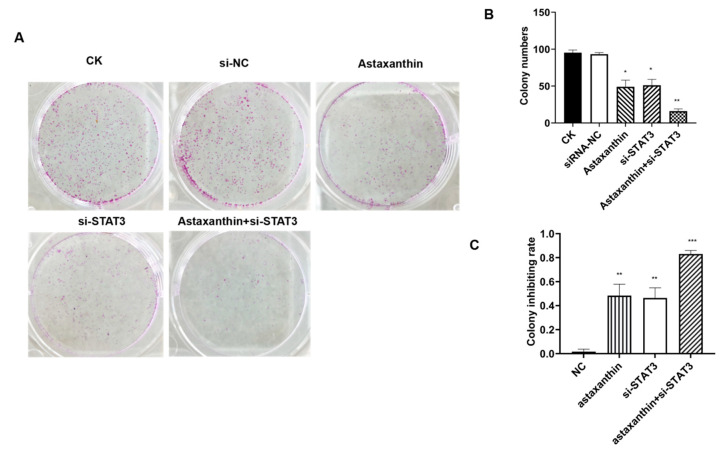
Colony inhibition rate analysis. The experiments were conducted on five groups. CK was the blank control group. The astaxanthin group was pretreated with astaxanthin for 24 h. The si-STAT3 group was transfected with siRNA-STAT3 for 48 h, and the siRNA-NC group was the blank control to si-STAT3. Astaxanthin+si-STAT3 group was transfected with a si-STAT3 plasmid for 48 h and treated with astaxanthin for 24 h. (**A**) Clone formation experiments were carried out. (**B**,**C**) Colony numbers were measured, and colony inhibition rates were calculated. The results show that astaxanthin inhibits the cloning ability of DU145 cells, while the effect of astaxanthin combined with si-STAT3 is superior. Data are presented as the average of three experiments (±SD), * p < 0.05, ** p < 0.01, *** p < 0.001.

The colony formation assay revealed that astaxanthin, siRNA-STAT3 and astaxanthin+siRNA-STAT3 reduced the number of colonies of DU145 cells (Figure 2A,B). The colony inhibition rates of astaxanthin, siRNA-STAT3 and astaxanthin+siRNA-STAT3 treatments were 48, 46 and 83%, respectively. Therefore, the results suggest that astaxanthin effectively inhibits the cloning ability of DU145 cells, while the effect of astaxanthin+si-STAT3 was as high as 83% (Figure 2C).

### 2.3. Astaxanthin Induces Apoptosis on DU145 Cells

**Figure 3:**
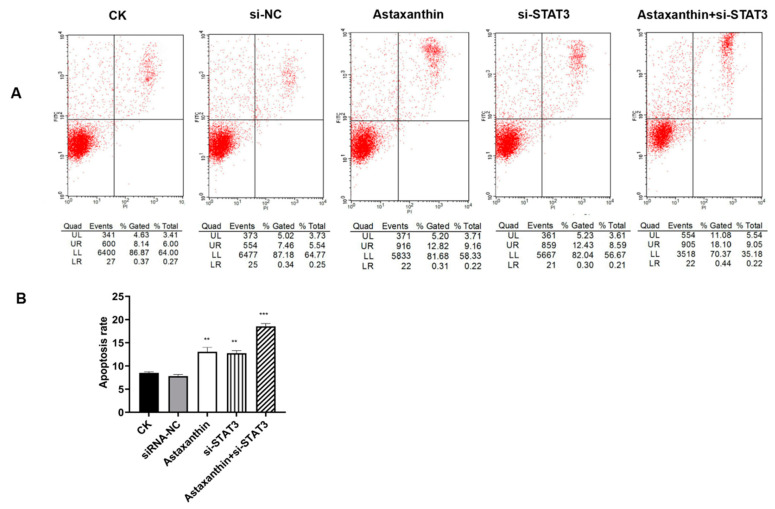
Effects of astaxanthin on apoptosis in DU145 cells. The experiments were divided into five groups. CK was the blank control group. The astaxanthin group was pretreated with astaxanthin for 24 h. The si-STAT3 group was transfected with siRNA-STAT3 for 48 h, and the siRNA-NC group was the blank control to si-STAT3. The astaxanthin+si-STAT3 group was transfected with a si-STAT3 plasmid for 48 h and then treated with astaxanthin for 24 h. (**A**) The progression of the apoptotic cells and the respective phase were analyzed with Annexin V-FITC assay. (**B**) The apoptosis rate was analyzed. The results show that both astaxanthin and si-STAT3 promote the apoptosis of DU145 cells, while the effect of astaxanthin combined with si-STAT3 is enhanced. Data are presented as the average of three experiments (±SD), ** p < 0.01 and *** p < 0.001.

To determine whether astaxanthin induces apoptosis, we used Annexin V-Fluorescein and a PI double staining assay. The experiments were divided into five groups: CK (blank control), astaxanthin, siRNA-NC (blank control to si-STAT3), si-STAT3 and astaxanthin+siRNA-STA3 (Figure 3A).

As shown in Figure 3B, compared to the CK group, treatment of DU145 cells with 200 μM astaxanthin for 24 h increased the percentage of apoptotic cells from 8.5% to 13.1%. The group containing si-STAT3 increased the percentage of apoptosis to 12.7%. However, the percentage of apoptosis was highest at 18.5% when combining astaxanthin with si-STAT3.

### 2.4. Astaxanthin Decreases the Migration and Invasion of DU145 Cells

**Figure 4:**
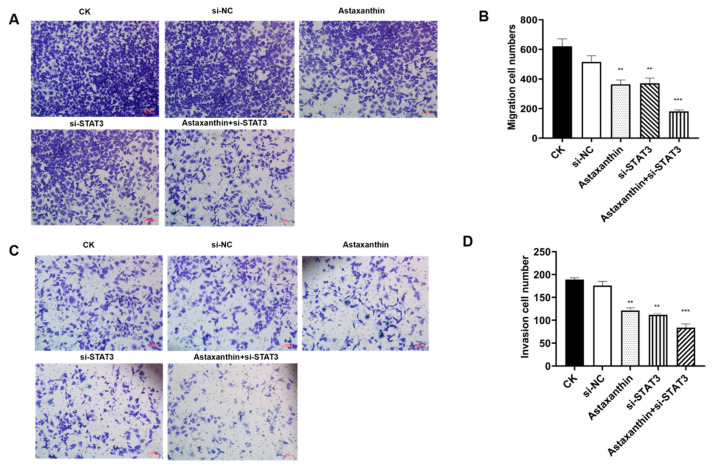
Astaxanthin influences the migration and invasion of DU145 cells. The groups are as described in Figure 2. (**A**) Image of the migratory transwell assay. (**B**) Both astaxanthin and si-STAT3 suppress the migration of DU145 cells, while the effect of astaxanthin combined with si-STAT3 is superior. Data are presented as the average of three experiments (±SD), ** p < 0.01 and *** p < 0.001. (**C**) Image of the invasive transwell assay. (**D**) Both astaxanthin and si-STAT3 suppress the invasion of DU145 cells, while the effect of astaxanthin combined with si-STAT3 is enhanced. Data are presented as the average of three experiments (±SD), ** p < 0.01 and *** p < 0.001.

Excessive migration and invasion are critical characteristics of tumor cells. We analyzed changes in the migration and invasion of DU145 cells with astaxanthin, si-STAT3 and astaxanthin combined with si-STAT3. As shown in Figure 4, astaxanthin and si-STAT3 decreased the migration and invasion of DU145 cells. When cells were treated with astaxanthin, about 41% of cells could not pass from one chamber to another (*p* < 0.01), and 36% cells could not pass through the transwell membrane as compared to the control group. When cells were treated with si-STAT3, the migration inhibition rate was 40%, and the invasion inhibition rate was 34%. In addition, the combination of astaxanthin and si-STAT3 increased the migration and invasion rates to 71% and 56% respectively.

### 2.5. Astaxanthin Reduces the Expression of STAT3 and the Combination of Astaxanthin and Silent STAT3 Increases the Reducing Effects

**Figure 5:**
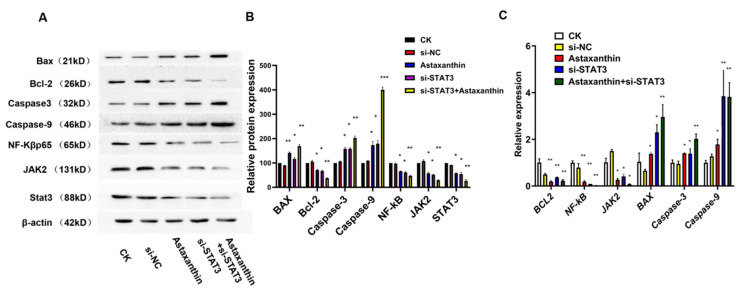
Astaxanthin influences the expression of STAT3. mRNA expression was consistent with that of the protein. (**A**) Western blotting results showed that astaxanthin suppresses the expression of STAT3 and the related proteins JAK2, NF-Kβp65 and Bcl-2, and it increases the levels of Caspase9, Bax and Caspase3. (**B**) Relative protein expression analysis. * p < 0.05, ** p < 0.01, and *** p < 0.001. (**C**) RT-PCR also confirmed that astaxanthin suppresses the expression of STAT3 and the related genes JAK2, NF-κBp65 and Bcl-2, and it increases the expression of Bax, Caspase3 and Caspase9. * p < 0.05 and ** p < 0.01.

To explore whether the inhibitory effects produced by astaxanthin were due to the STAT3-related pathway, we examined the expression of JAK2, Caspase3, Caspase9, NF-κB, BAX and BCL-2. As shown in Figure 5A, astaxanthin downregulates the protein expression of JAK2, BCL-2 and NF-κB and upregulates the protein expression of BAX, Caspase3 and Caspase9. These results indicate that the effects of inhibiting proliferation, increasing apoptosis and weakening migration and invasion of astaxanthin could reduce the expression of STAT3 and the related pathway proteins. Thus, the combination of astaxanthin and si-STAT3 improved the anti-tumor effects.

RT-PCR (Figure 5B) analyses also confirmed that anti-tumor effects by astaxanthin were due to the suppression function of STAT3 and the related pathway gene. Astaxanthin downregulates the gene expression of JAK2, BCL-2 and NF-κB and upregulates the gene expression of BAX, Caspase3 and Caspase9.

This profile revealed that the lower expression of STAT3 caused by astaxanthin effectuated anti-tumor functions.

### 2.6. Impact of Astaxanthin on the Growth of Xenograft Tumors

**Figure 6:**
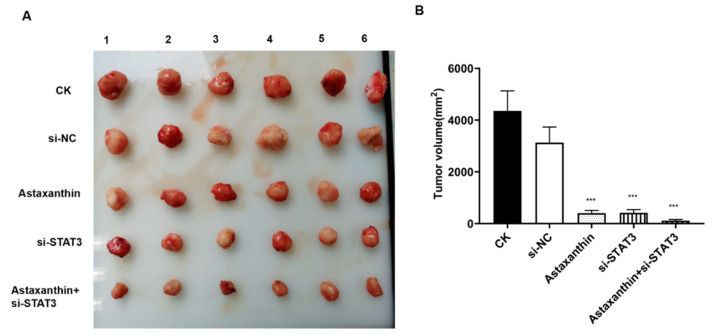
Astaxanthin inhibits the growth of DU145 tumor xenografts in nude mice. (**A**) Representative observations of the growth of the five groups. (**B**) Both astaxanthin and si-STAT3 effectively compress the tumor growth, while the effect of astaxanthin combined with si-STAT3 is superior. Data are presented as the average of three experiments (±SD), *** p < 0.001.

Next, we explored whether the inhibitory effects of astaxanthin were operative in xenograft tumors. Five xenograft models were established in nude mice. Both astaxanthin and si-STAT3 suppressed the growth of tumors. These suppression effects were enhanced when astaxanthin and si-STAT3 were combined (Figure 6A,B), supporting the cell-based observation that astaxanthin exerts a significant inhibitory effect on tumor growth. These results described the effect of astaxanthin by suppressing the expression of STAT3, thereby resulting in the inhibition of xenograft tumors and confirming the hypothesis derived from cell-based experiments.

## 3. Discussion

Herein, we used aggressive prostate cancer DU145 cells and found that astaxanthin inhibits their proliferation and suppresses the expression of STAT3. In addition, the cloning ability of the cells can be reduced. Interestingly, astaxanthin promotes apoptosis of DU145 cells, while the ability of migration and invasion is reduced. The combination of astaxanthin and si-STAT3 exhibits a pronounced reduction effect.

Prostate cancer is a malignant tumor of the urogenital system in elderly men, and the incidence and death rate of the disease are rising. In the USA and European countries, prostate cancer is the highest incidence of malignant tumors and the second leading cause of mortality. In China, the incidence of prostate cancer also shows a clear upward trend [1,2]. Primary treatments for prostate cancer include radical surgery, hormone therapy, radiotherapy and chemotherapy. However, resistance to these treatments remains a poorly elucidated phenomenon rendering the tumor excessively aggressive [3,4,5]. Therefore, finding a drug that can effectively suppress aggressive tumor cells is clinically crucial.

Accumulating evidence demonstrates that STATs play a major role in the process of tumor formation, especially STAT3 [35,36]. Activated STAT3 is involved in tumorigenesis by regulating multiple pathways, such as apoptosis suppressor genes (Mcl-l, Bcl-x L and Survivin), cell cycle regulators (Cyclin Dl/D2 and C-myc), angiogenesis factors (vascular endothelial growth factor (VEGF)) and tumor metastasis-related genes (matrix metalloproteinases (MMPs)) [37,38]. STAT3 is an oncogene with functional activation closely related to the occurrence of prostate cancer. In addition, it shows abnormal expression or increased activity in human leukemia, multiple myeloma, squamous cell carcinoma of the head and neck, breast cancer and prostate cancer. This signaling pathway has been confirmed as an effective molecular target for various human tumor interventions [39,40]. Several molecules of the STAT3 pathway, especially the genes regulated by STAT3, are related to the occurrence and development of tumors. Hence, treating cancer by targeting STAT3 via inhibition of the activation or expression of the molecule, to block the signal transmission pathways of the tumor cells, seems an optimal approach.

About 67% of anti-cancer drugs are derived from natural products or natural product derivatives [16], while >200 natural product-derived drugs are in the preclinical or clinical development stage [41]. In recent years, several studies have reported that natural products and their derivatives, such as alantolactone and 6,7-dimethoxycoumarin, affect the progress of tumors or inflammation by regulating the protein activity or transcription level of the STAT3 signaling pathway. These findings provide new directions for the discovery of new drugs [42,43,44,45]. Although it has been shown that many STAT3 inhibitors have anti-tumor effects in vitro, screening out inhibitors with high efficiency, low toxicity and fewer side effects is rather challenging, and further animal experiments have studied less in relation to the pharmacology and toxicology of related inhibitors. Currently, only a few inhibitors are under clinical evaluation [46,47,48]. Therefore, inhibitors with high efficiency, low toxicity and fewer side effects need to be developed urgently.

Astaxanthin is a natural product that is safe for use and has anti-cancer properties. However, whether it can suppress prostate cancer through STAT3 is not yet elucidated. In the current study, we found that astaxanthin inhibits the proliferation of DU145 prostate cancer cells by reducing the level of STAT3.

Nevertheless, the present study has several limitations. First, in addition to inhibiting levels of STAT3, there may be other mechanisms of the inhibiting effects of astaxanthin on tumor cells. In this study, we explored only STAT3 and the related pathways, thereby necessitating the analysis of other participating genes. Second, we explored the inhibiting effects of astaxanthin on aggressive prostate cancer cells. However, whether it can reduce the resistance to radiotherapy or chemotherapy necessitates further studies.

**Figure 7:**
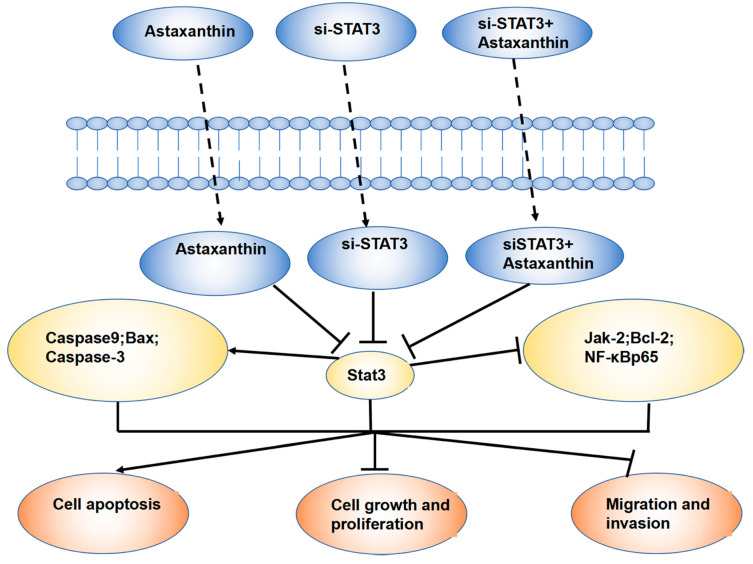
Schematic representation of the mechanism of action of astaxanthin on prostate cancer DU145. Astaxanthin inhibits DU145 tumor cells by reducing the levels of STAT3 and its related proteins. When astaxanthin and si-STAT3 are combined, the effects are better.

Furthermore, astaxanthin was found to inhibit proliferation and cloning formation, promote apoptosis and weaken the invasion and migration ability of DU145 cells. Western blotting and RT-PCR confirmed that astaxanthin not only reduces the expression of STAT3, but also that of the related molecules at protein and mRNA levels. Together, the effects described above were significant when astaxanthin was combined with si-STAT3. The results of animal experiments were consistent with those of cell experiments (Figure 7).

## 4. Materials and Methods

### 4.1. Cell culture and Reagents

The DU145 prostate cancer cell line was provided by the Urinary Surgery Department of the First Affiliated Hospital of Peking University and cultured in RPMI 1640 medium (M&C Gene Technology, Beijing, China) supplemented with 10% fetal bovine serum (FBS, Gibco, Auckland, New Zealand). The cells were maintained in a humidified incubator with 5% CO_2_ at 37 °C. Astaxanthin was purchased from Sigma–Aldrich (St. Louis, MO, USA).

### 4.2. MTT Assay

A density of 103–104 DU145 cells/well were seeded into 96-well plates. A volume of 100 μL fresh culture medium containing different concentrations of drugs was added and incubated for 48 h, following which 20 μL of MTT solution (5 mg/mL in phosphate-buffered saline (PBS), pH = 7.4) was added to each well, and incubation continued for an additional 4 h. Finally, 150 μL DMSO was added to each well, and the absorbance was measured at 490 nm on the ELISA reader (Cayman Chemical, Ann Arbor, MI, USA).

### 4.3. Colony Assay

Drug treatment: The corresponding groups were transfected with siRNA-STAT3 and siRNA-NC, respectively, and 48 h post-transfection, 200 μM astaxanthin drug was administered for 24 h.

Clone formation: The culture was continued for 10–14 days. The cells were then harvested in PBS, fixed with 4% paraformaldehyde for 20 min and stained with 0.1% crystal violet for 20 min.

### 4.4. Flow Cytometry Assay

Annexin V-FITC/PI assay was used. The cells from each group were harvested and stained with 5 μL Annexin V-FITC/PI staining solution in the dark for 5 min. The rate of apoptosis was detected by flow cytometry (Columbus 2.4, PerkinElmer, Waltham, MA, USA).

### 4.5. Cell Migration and Cell Invasion Assay

Detection of cell migration: The cells of each group were collected, resuspended in 2% serum medium and inoculated in the upper transwell chamber, while 10% serum was added to the lower chamber. The cells were fixed with 4% paraformaldehyde after 16 h of incubation. Subsequently, the cells in the upper layer of the chamber membrane were wiped with a cotton swab, stained with crystal violet for 20 min, washed twice with PBS, dried, and images were then captured.

Detection of cell invasion: The cells of each group were collected, resuspended in 2% serum medium and inoculated in the upper transwell chamber (the upper chamber pretreated with Matrigel), while the lower chamber was supplemented with 10% serum medium. After 16 h of culture, 4% paraformaldehyde was used to fix the cells, the upper cell membrane was wiped with a cotton swab, stained with crystal violet for 20 min, washed twice with PBS, and images were acquired after drying.

### 4.6. Western Blot

Protein expression in DU145 cells treated with either CK (control) or si-NK, astaxanthin or si-STAT3, or astaxanthin and si-STAT3 was evaluated using Western blotting. DU145 cells were seeded in 6-well plates and cultured to 80% confluency. The cells were then treated with CK (control) or si-NK, astaxanthin or si-STAT3, or astaxanthin and si-STAT3 for 24 h, followed by protein extraction. An equivalent of 30 μg protein was subjected to SDS-PAGE and Western blotting. The primary antibodies were diluted as follows: Bcl-2(26 kDa) 1:1000 (ab692, Abcam, Cambrige, MA, USA), Bax (21 kDa) 1:1000 (ab7977, Abcam, Cambrige, MA, USA), Caspase3 (32 kDa) 1:1000 (ab32351, Abcam, Cambrige, MA, USA), Caspase9 (46 kDa) 1:1000 (ab32539, Abcam, Cambrige, MA, USA), NF-κB p65 (65 kDa) 1:1000 (ab76302, Abcam, Cambrige, MA, USA), JAK2 (131 kDa) 1:1000 (ab92552, Abcam, Cambrige, MA, USA) and Stat3 (88 kDa) 1:1000 (ab119352, Abcam, Cambrige, MA, USA). Each Western blotting assay was repeated at least two times.

### 4.7. RT-PCR

Total RNA was extracted using Baosai lysis reagent (Baosai, Hangzhou, China; RE02050), and 1 μg was reverse-transcribed using the RevertAid First Strand cDNA synthesis kit (Baosai; RT02020), according to the manufacturer’s instructions. For qRT-PCR, 25–50 ng of cDNA was used as the template for PCR amplification using the fluorescence quantification kit (Baosai; PM10003). Primers for PCR amplification were as follows: Bcl2-F: CCCCGTTGCTTTTCCTCTG; Bcl2-R: CATCACTATCTCCCGGTTATCG; Bax-F: TTTCCGAGTGGCAGCTG; Bax-R: CAAAGTAGAAAAGGGCGACAAC; Casp3-F: CCCATTTCCTACAGAACGACC; Casp3-R: CATCAATGAATCTAAAGTGCGGG; Casp9-F: CCTAGAAAACCTTACCCCAGTG; Casp9-R: TCAAGAGCACCGACATCAC; NF-κB-F: ACCCTGACCTTGCCTATTTG; NF-κB-R: GCTTGGCGGATTAGCTCTTT; JAK2-F: AGTAAAGATGCCTTCTGGTGAA; JAK2-R: TCCATTTCCAAGTTCTCCACT; STAT3-F: GGGACACTGGGTGAGAGTTA; STAT3-R: CACACACACACAAGCCATC; actin-F: GGTGGTCTCCTCTGACTTCAACA; actin-R: GTTGCTGTAGCCAAATTCGTTGT.

### 4.8. Construction of DU145 Tumor Model

In the first group, DU145 cells were cultured up to the logarithmic phase, and 2 × 10^7^ cells were inoculated in the left armpit of each of 20 nude mice. In the second group, 10 inoculated nude mice were transfected with blank vector for 24 h, while the third group of cells was transfected with the interference vector si-STAT3. At 24 h post-interference, 20 nude mice were inoculated. After 2 weeks of inoculation and culture, 12 tumor-bearing nude mice were randomly selected from the first group and divided into two groups. Six were given saline and the other six were given astaxanthin solution. In the second group, six tumor-bearing nude mice were selected for continuous culture. In the third group, 12 tumor-bearing nude mice were randomly selected and divided into two groups: six were given intragastric astaxanthin solution and the other six were continued in culture.

Group A: Blank control group, intragastric administration of normal saline; Group B: Blank transfection group; Group C: Astaxanthin at a dose of 200 mg/kg administered intragastrically once a day. Nude mice weighed approximately 20 g, the drug concentration was 40 mg/mL, and 100 μL suspension was administered by gastric perfusion; Group D: Interference group; Group E: Interfered with the astaxanthin gavage group. Nursing mice were sacrificed after continuous gavage for 3 weeks, tumor tissues were removed and weighed and images captured.

### 4.9. Ethics Statement

All experiment procedures were conducted in strict accordance with the appropriate institutional guidelines for animal research. The protocol was approved by the Committee on the Ethics of Animal Experiments of Beijing Union University. The approval number is: CIHFBUU-ZYZDS-B01-15-01.

### 4.10. Statistical Analysis

The Student’s *t*-test was used to determine the statistical difference between the means of two groups and data. A *p*-value < 0.05 indicated statistical significance.

## 5. Conclusions

Astaxanthin can effectively inhibit the proliferation and cloning formation of DU145, promote the apoptosis of the DU145 cells and weaken the invasion and migration ability of DU145. In addition, astaxanthin can not only reduce the expression of STAT3 but also the expression of the related proteins of STAT3 in protein and mRNA levels. When astaxanthin and si-STAT3 were combined, all the effects were improved. The results of animal experiments are consistent with the results of cell experiments.

Thus, we concluded that astaxanthin inhibits DU145 tumor cells by reducing the level of STAT3. It may be a prospective drug that can effectively suppress aggressive tumor cells.
